# Variation in the dynamic of absorption and efficiency of phosphorus use in tomato

**DOI:** 10.1038/s41598-022-08337-3

**Published:** 2022-03-14

**Authors:** Douglas José Marques, Ernani Clarete da Silva, José Andrés Carreño Siqueira, Elham Abedi, Fernanda Rosa Veloso, Gabriel Mascarenhas Maciel, Wilson Roberto Maluf

**Affiliations:** 1 Institute of Agricultural Sciences, Federal University of Uberlândia - UFU, Rodovia LMG 746, Km 01, Bairro Araras, Monte Carmelo, MG Brazil; 2 Agricultural Sciences, Federal University of São João del-Rei - UFSJ, Sete Lagoas, MG Brazil; 3 Department of Biology, Science and Research Branch, Islamic Azad University, Tehran, Iran; 4 Department of Agriculture, Federal University of Lavras - UFLA, Lavras, MG Brazil

**Keywords:** Plant sciences, Plant stress responses, Abiotic

## Abstract

Changes in root growth and metabolism of P in tomato cultivars are necessary in acidic soils in tropical and subtropical regions in response to P deficiency. This increase in the efficiency of phosphorus absorption by tomatoes can significantly reduce the doses of phosphate fertilizers used, as well as, possibly, the more immediate use of P fixed in the soil matrix, with favorable effects on agricultural sustainability, promoting the use of marginal areas in terms of soil fertility, and the national fertilizer economy. The tested hypothesis was that there would be no difference in the uptake and utilization of P by tomato cultivars; therefore, this study investigated the variation in the dynamics of absorption and efficiency of P-use through changes in the root, stem, leaf, gas exchange, and P-use efficiency in tomato cultivars contrasting P-absorption. The experimental design comprised a factorial scheme consisting of two cultivars that were tolerant and sensitive to P deficiency and three P concentrations (control, moderate deficiency, and severe deficiency). P limitation in the tolerant cultivar promoted high dry matter concentration (root, stem, and leaf), leaf area, root volume, nutrient translocation, rate of leaf gas exchange, and efficiency under P-deficiency stress. It was concluded from the research that the variation in the dynamics of absorption and efficiency of P use of the tolerant cultivar increased the production of roots, leaves, and leaf gas exchange under P stress conditions.

## Introduction

Phosphorus (P) is an essential element that participates in several plant metabolic processes. Global food security is dependent on the use of fertilizers. This dependence occurs especially with phosphates made from a limited amount of phosphate ore. In addition, the worldwide supply of P for the manufacture of fertilizers is a non-renewable natural resource that requires the conscious use of this nutrient to ensure the sustainability of agriculture in its current form^1^. In tropical areas, P deficiency has become one of the principal limiting factors for crop growth due to the loss of P nutrients caused by high temperatures and the fixation of P by iron and aluminum oxides in the soil^2^. More efficient methods for using fertilizers and finding ways to recover phosphorus are needed. Therefore, flexibility in plant metabolism^3^, in response to P limitation, plants have evolved various biochemical, metabolic, and morphological adaptations to enhance P acquisition^2^.

The increase in the world's population and the expanding global affluence creates an increased demand for more food, particularly meat, which increasingly places more strain on the accessible supply of phosphorus^4^. The estimated amount of phosphorus that people consume is only one-fifth of that mined, suggesting that significant amounts are simply being released into the environment. Agriculture is by far the main consumer of mined phosphorus worldwide, accounting for between 80 and 90% of the world’s total demand^5^.

Brazil is critical to food production and global food security, but its soils require inorganic phosphorus (P) fertilizers for agricultural production (2.2 Tg increasing to 4.6 Tg in 2050)^6^. Fertilizers have been widely used in many intensive cropping systems^7^ because of their low concentration in soils (0.5–5 g kg^−1^), but only a small fraction is available for plants^8^. In addition, crops can use only 10–30% of the phosphate within the fertilizer, usually in each application year^9^.

Enhancing P efficiency has long been a challenge in intensive cropping systems^10^. More extensive root systems increase the contact area between the roots and the soil and are able to absorb most of the phosphorus fertilizer that remains in the soil^11^. The demand and low availability of rock phosphate as a source of P fertilizer and the increasing awareness of the negative environmental consequences of high P fertilizer input have also increased the interest in improving the efficiency of P acquisition and utilization by plants^12^.

One alternative to traditional methods aimed at reducing phosphorus intake is the development of tomato cultivars with fine roots in large amounts and achieve efficient metabolism of P. Root hairs are extensions occupying up to 90% of a root's surface, and they facilitate water and nutrient acquisition^13^. Root hairs have an ideal geometry for P-capture because their small radius helps to reduce the carbon cost to the plant while also extending soil exploration^14^. Furthermore, root hairs are fine, which helps in P acquisition. A plant growth model based on P-deficient rice revealed that increasing root fineness by 22% increased P uptake by three-fold^15^.

The increase in the efficiency of phosphorus absorption by the tomato plant could provide a significant reduction in the doses of phosphate fertilizers used, as well as lead to more immediate utilization of the P fixed in the soil matrix. This would bring favorable strides towards agricultural sustainability and improve the net income of the rural producer, the use of marginal areas, and the fertilizer economy on the national level. The tested hypothesis was that there would be no difference in the absorption and use of P in tomato cultivars. In addition, a crop’s reliance on acquisition efficiency and internal P-utilization efficiency is thought to be mostly under genotypic control, and differs among crop species and genotypes within the same species^16^. Variation in P acquisition among genotypes was strongly correlated with root length^17^. Consequently, root growth, leading to increased root-soil contact, is an important factor determining the uptake of less mobile nutrients, such as phosphorus, in soils^18^. The selection of root hairs may be a feasible option for improving the P-acquisition of crop plants^19^. Root hairs can exploit soil near the root surface more effectively because of their geometric arrangement on roots and their ability to increase root surface area^20^. Previous studies^21^ have shown the importance of root hairs for P uptake through differences between plant species. However, there are few reports on whether variations in root hair formation exist between genotypes.

Tomato introduction with greater efficiency in phosphorus absorption has been identified^22^. This genotype screening involved more than 200 accessions of tomato (*Solanum lycopersicon*), with at least two introductions highly efficient in the extraction of P from phosphorous-poor nutrient solutions. In one of these introductions (cultivar “Globonnie”), the efficiency of phosphorus extraction was associated with a morphological characteristic associated with roots when the accession was cultivated in a nutrient solution with low P concentration. This characteristic, called "cottony root,” was shown to be of simple inheritance (a recessive gene, called *crt*) and is associated with a large number of radicels or hair roots, which can be observed under a microscope after staining with acetic carmine when plants grow in solutions with low (2 mg L) P concentration^22^. This response was not observed when higher P concentrations (8 mg L) were used^22^. The availability of tomato germplasm with efficiency in phosphorus extraction is associated with a morphological characteristic (large number of roots or root hairs when cultivated under low levels of P) and the simple inheritance of this characteristic controlled by a recessive gene^22^, making this species particularly suitable for genetic improvement aiming at greater phosphorus absorption.

This study investigated the variation in the dynamics of absorption and efficiency of P-use through changes in the root, stem, leaf, gas exchange, and P-use efficiency in tomato cultivars contrasting P-absorption.

## Results

### Mass from the dry matter of tomato cultivars in P stress

**Figure 1:**
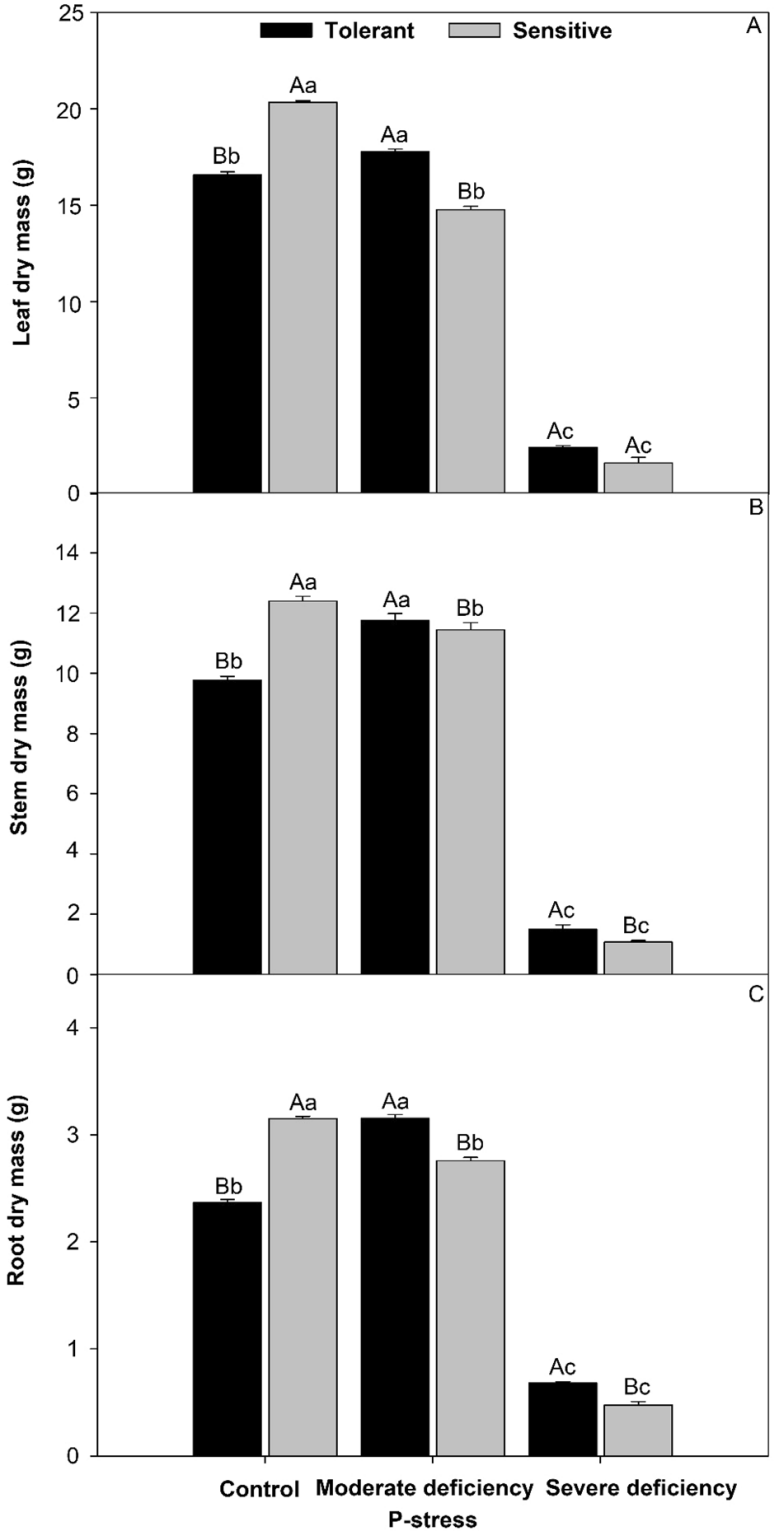
Dry mass of the tomato plants leaves (**A**), stems (**B**), and root (**C**) in cultivars tomato treated P-stress. Columns with different capital letters compare cultivars (tolerant and sensitive) between P stress levels (control, moderate deficiency, and severe deficiency). Lowercase letters compare cultivars (tolerant and sensitive) with the same color as a function of P stress levels (control, moderate deficiency, and severe deficiency). Different letters differ by the Scott-Knott test (P < 0.05). Columns corresponding to means of six repetitions and standard deviations.

**Figure 2:**
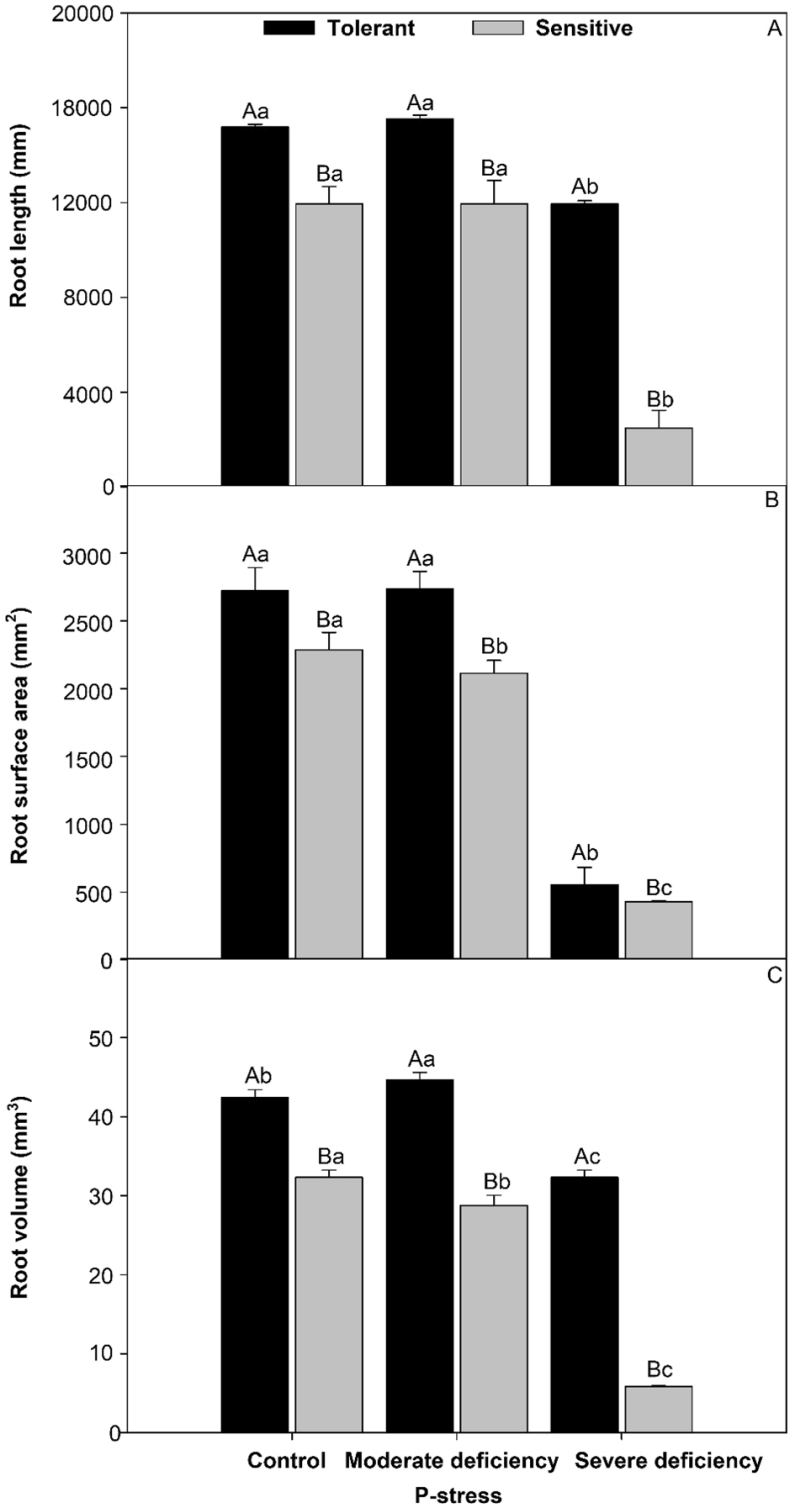
Root length (**A**), root superficial area (**B**), and root volume (**C**) in cultivars tomato treated P-stress. Columns with different capital letters compare cultivars (tolerant and sensitive) between P stress levels (control, moderate deficiency, and severe deficiency). Lowercase letters compare cultivars (tolerant and sensitive) with the same color as a function of P stress levels (control, moderate deficiency, and severe deficiency). Different letters differ by the Scott–Knott test (P < 0.05). Columns corresponding to means of six repetitions and standard deviations.

The cultivar that was sensitive to P stress produced higher dry matter concentration in the leaf (8.1%) (Fig. 1A), stem (7.9%) (Fig. 1B), and root (7.4%) (Fig. 2C) than in the control. Under moderate deficiency, the tolerant cultivar increased the production of dry matter by 8.3% in the leaf, 10.0% in the stem, and 8.7% in the roots. Under severe deficiency, the tolerant cultivar presented higher values than the control (7.0% in the leaf, 8.0% in the stem, and 7.0% in the root).

### Root production in P-deficiency stress

Regardless of the levels of P, the tolerant cultivar had an increase of 1.76% in root length (Fig. 2A), 14.28% in root surface area (Fig. 2B), and 13.0% in root volume (Fig. 2C) compared to the sensitive cultivar. It is important to note that under severe deficiency, the tolerant cultivar presented values much higher than those of the sensitive cultivar in root production.

### Different root sizes in P-deficiency stress

**Figure 3:**
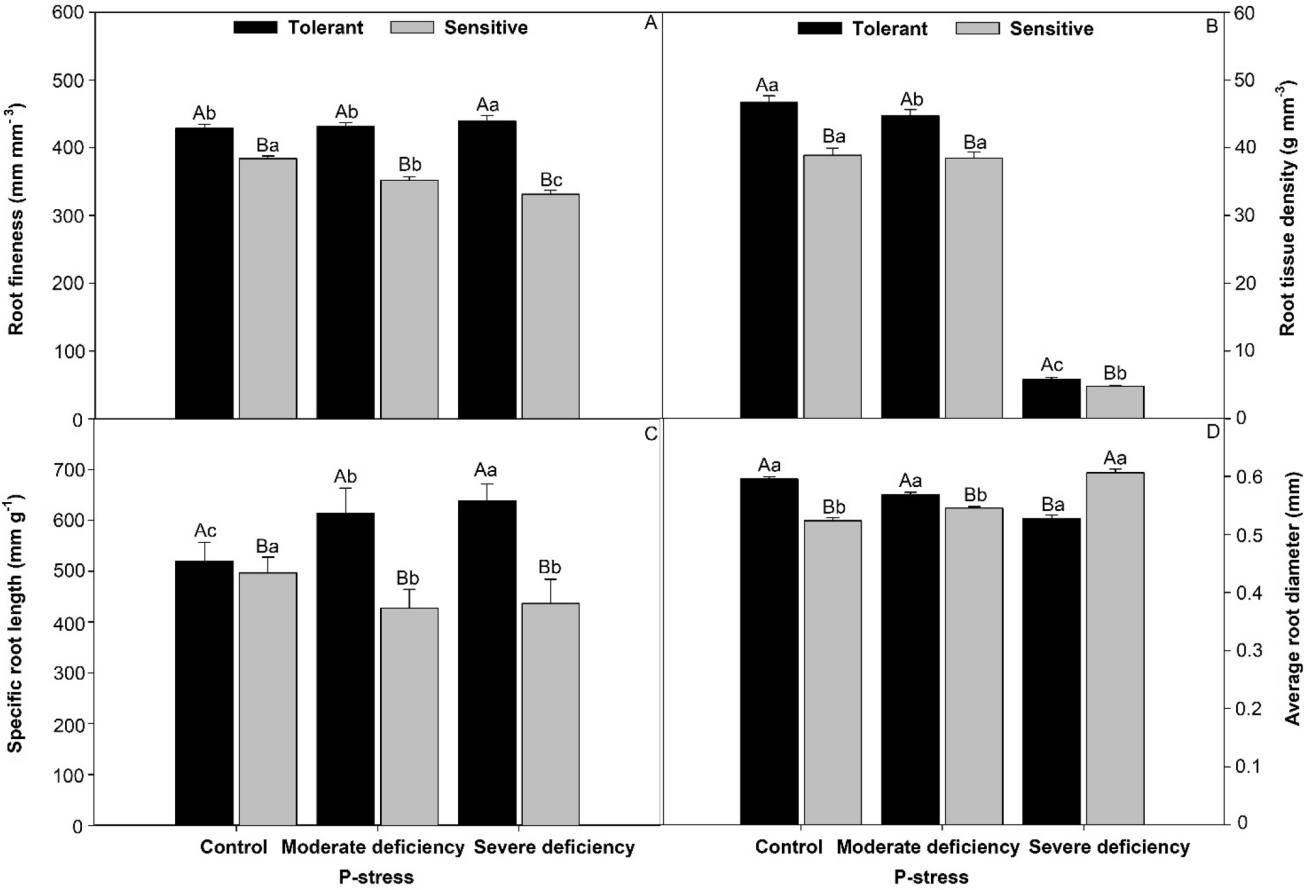
Finesse root (**A**), root tissue density (**B**), specific root length (**C**), and average root diameter (**D**) in cultivars tomato treated P-stress. Columns with different capital letters compare cultivars (tolerant and sensitive) between P stress levels (control, moderate deficiency, and severe deficiency). Lowercase letters compare cultivars (tolerant and sensitive) with the same color as a function of P stress levels (control, moderate deficiency, and severe deficiency). Different letters differ by the Scott–Knott test (P < 0.05). Columns corresponding to means of six repetitions and standard deviations.

The tolerant cultivar presented values that were 12% higher in root thinness (Fig. 3A) than those in the control treatment; these were 8.6% higher in moderate deficiency and 7.8% higher in severe deficiency. The specific length of the root (Fig. 3B) was higher for the tolerant cultivar; specifically, this was 10% higher than that in the control, 82% in moderate deficiency, and 67% in severe deficiency. As for root tissue density (Fig. 3C), the tolerant cultivar showed values that were 11% higher than those in the control, 8.0% higher in moderate deficiency, and 8.0% higher in severe deficiency. For root diameter (Fig. 3D), the tolerant cultivar had values that were 8.7% higher than those of the control, 8.5% in severe deficiency, and 9.6% in moderate deficiency.

### Translocation and P concentration in cultivars

**Figure 4:**
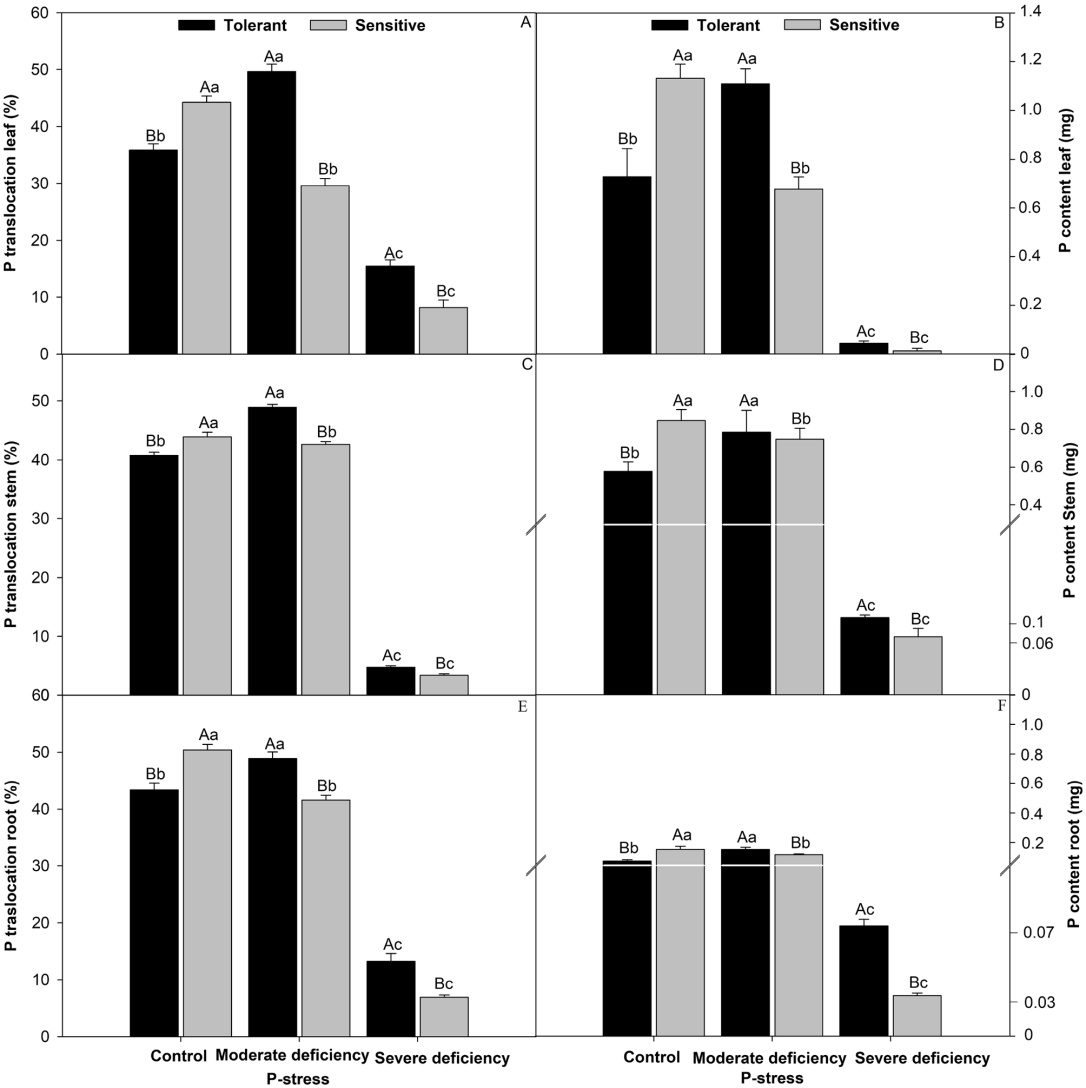
The P-translocation leaf (**A**), P-content leaf (**B**), P-translocation steam (**C**), P-content steam (**D**), P-translocation root (**E**), and P-content root (**F**) in cultivars tomato treated P-stress. Columns with different capital letters compare cultivars (tolerant and sensitive) between P stress levels (control, moderate deficiency, and severe deficiency). Lowercase letters compare cultivars (tolerant and sensitive) with the same color as a function of P stress levels (control, moderate deficiency, and severe deficiency). Different letters differ by the Scott–Knott test (P < 0.05). Columns corresponding to means of six repetitions and standard deviations.

The sensitive cultivar showed an increase of 8.1% compared with the control. As the stress level increased, the translocations in the specimen of the tolerant cultivar became superior, by 60% with moderate deficiency and 52% with severe deficiency (Fig. 4A). Regarding P translocation in the stem (Fig. 4B), relative to the control, the sensitive cultivar was higher by 9.2%; in the moderate and severe deficiency treatments, the tolerant cultivar was higher by 8.7% and 9.7%, respectively. For P translocation at the root (Fig. 4C), the sensitive cultivar was 8.6% higher, following the same trend as the tolerant cultivar, which was 8.5% higher under moderate deficiency and 52% under severe deficiency. The P concentration in the leaf (Fig. 4D) was 6.3% higher in the sensitive cultivar than in the control; it was also 60% higher under moderate deficiency and 3% under severe deficiency for the tolerant cultivar. In the stem, the P concentration (Fig. 4E) followed the same control sequence as the sensitive cultivar, which was 14.7% higher, whereas under moderate and severe deficiency, the tolerant cultivar was superior, yielding values that were 9.5% and 7% higher than the control. For the P concentration in the root (Fig. 4F), the sensitive cultivar had values 48% higher than in the control, while the increase in the P-stress values in the tolerant cultivar was higher under moderate deficiency (76%) and severe deficiency (36%). We observed that under P-stress conditions, the tolerant cultivar was more efficient in the use of P.

### Leaf area of tomato cultivars in different P levels

**Figure 5:**
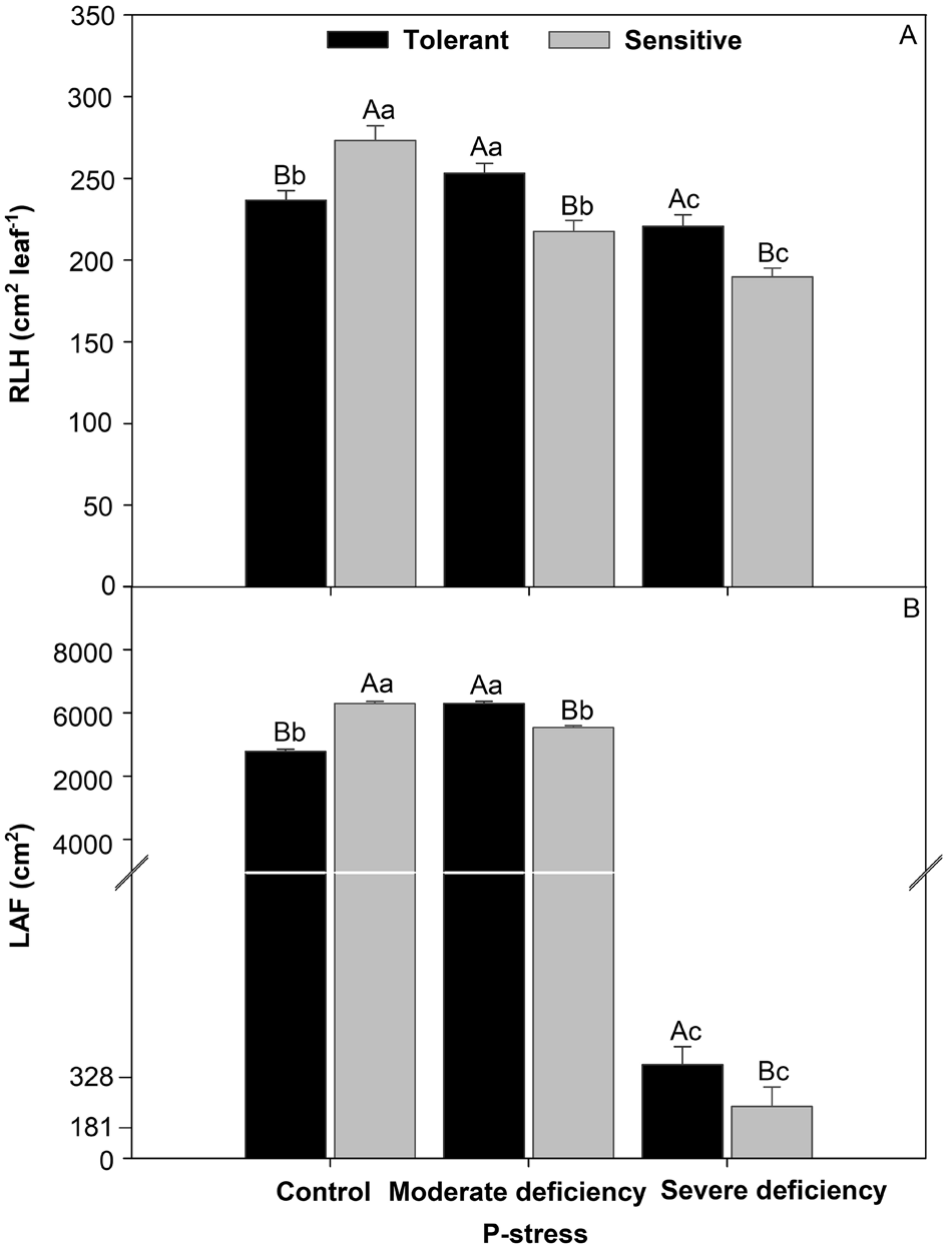
Relative leaf area (**A**) and leaf are (**B**) in tomato treated P-stress cultivars. Columns with different capital letters compare cultivars (tolerant and sensitive) between P stress levels (control, moderate deficiency, and severe deficiency). Lowercase letters compare cultivars (tolerant and sensitive) with the same color as a function of P stress levels (control, moderate deficiency, and severe deficiency). Different letters differ by the Scott-Knott test (P < 0.05). Columns corresponding to means of six repetitions and standard deviations.

The sensitive cultivar yielded values that were 10% higher than those of the control. Furthermore, with increased levels of P stress, the specific leaf area was higher for the tolerant cultivar, with 12% in moderate deficiency and 10% in severe deficiency (Fig. 5A). For the leaf area, the same trend was observed for the 76% CV control. Sensitive, with an increase in P stress, 87% moderate deficiency, and 55% severe deficiency in the tolerant cultivar (Fig. 5B).

### Leaf gas exchange of tomato plants in P stress

**Figure 6:**
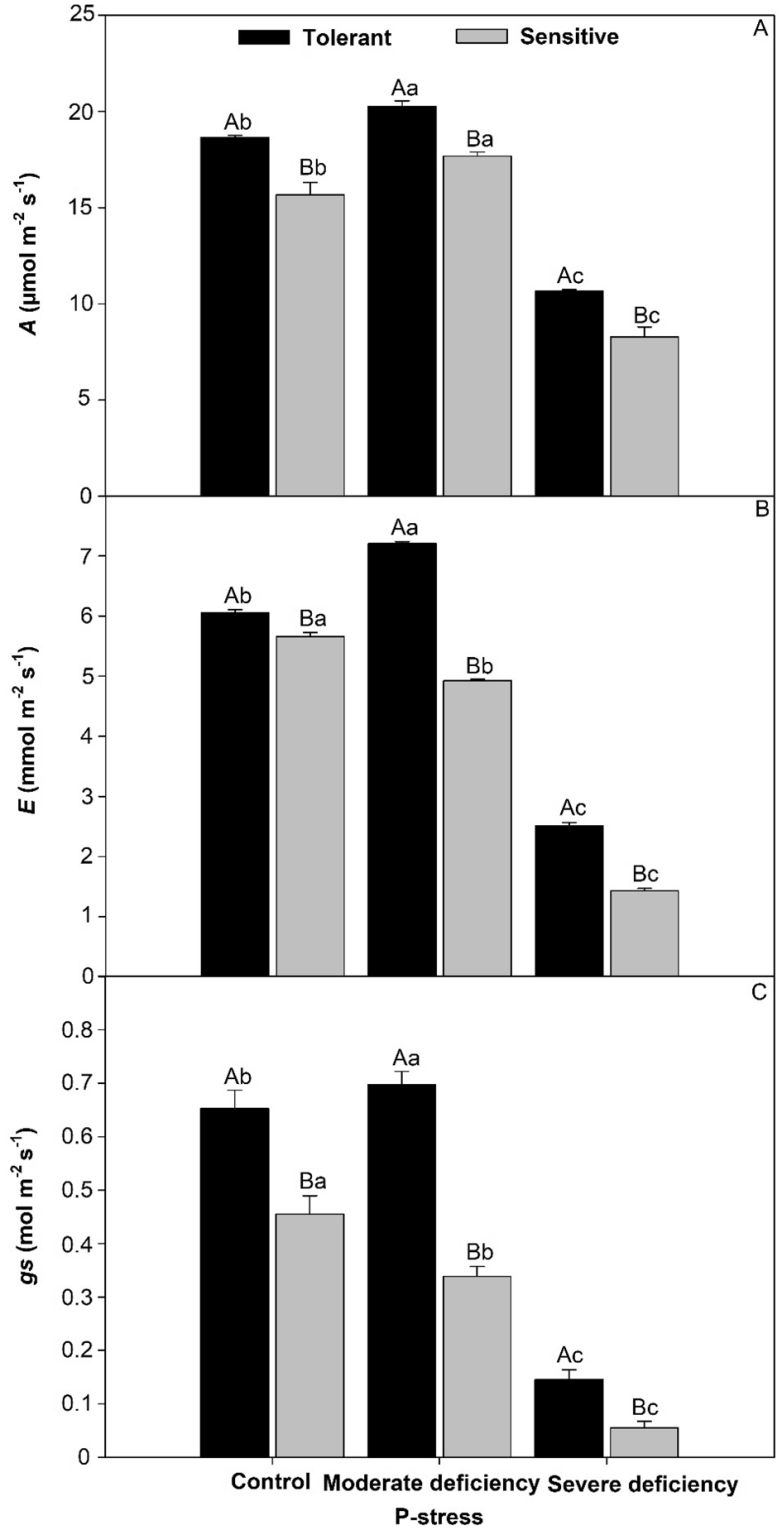
Net assimilation (**A**), transpiration rate (**B**), and stomatal conductance (**C**) in cultivars tomato treated P-stress. Columns with different capital letters compare cultivars (tolerant and sensitive) between P stress levels (control, moderate deficiency, and severe deficiency). Lowercase letters compare cultivars (tolerant and sensitive) with the same color as a function of P stress levels (control, moderate deficiency, and severe deficiency). Different letters differ by the Scott-Knott test (P < 0.05). Columns corresponding to means of six repetitions and standard deviations.

The net assimilation rate (Fig. 6A) was 3% higher in the sensitive cultivar than in the control, 9% higher under moderate deficiency, and 10% higher under severe deficiency for the tolerant cultivar. For transpiration and stomatal conductance (Fig. 6B,C), the tolerant cultivar had values 20% and 50% higher, respectively.

### P efficiency in deficiency stress levels

**Figures 7:**
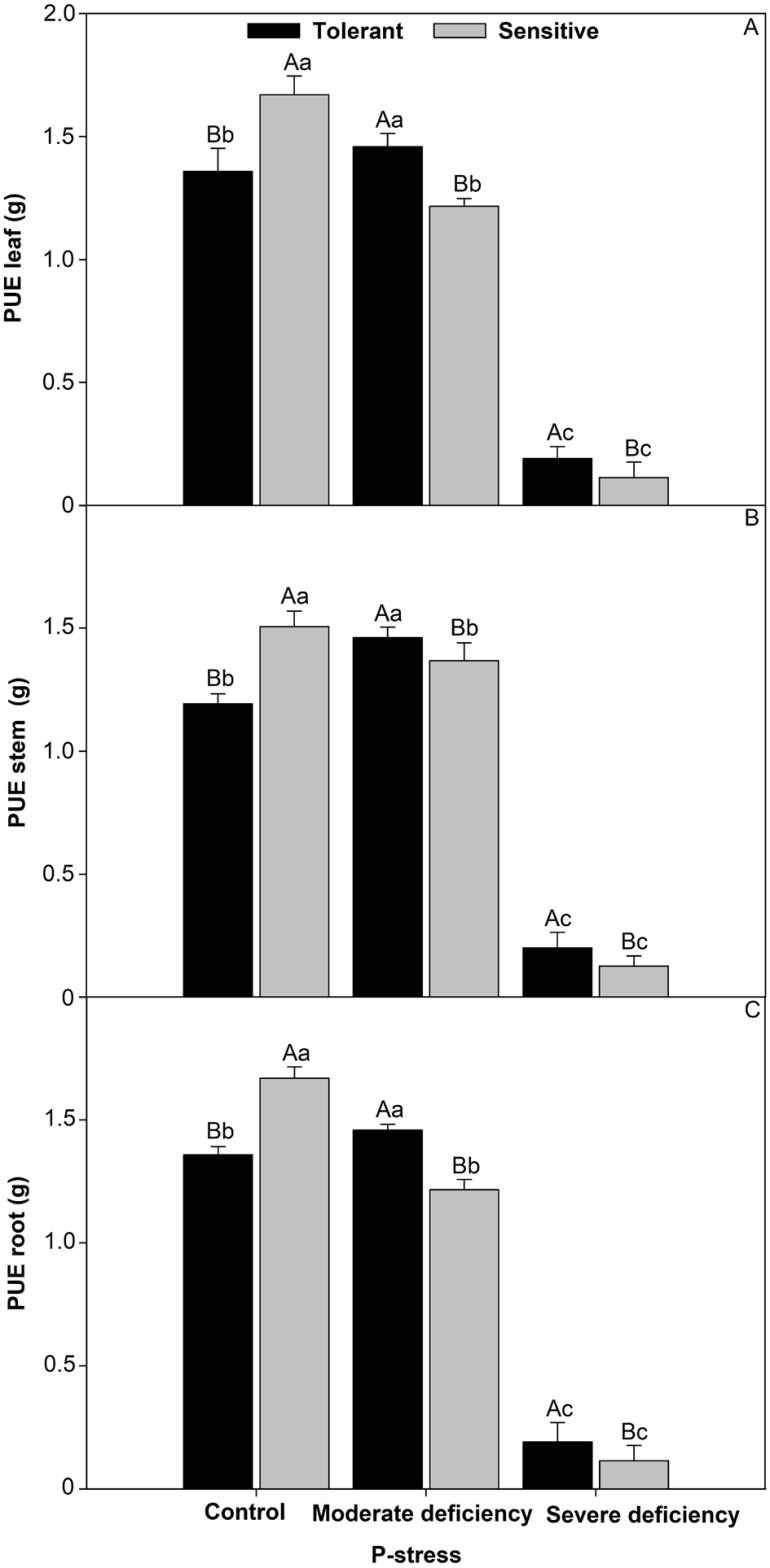
P-use efficiency leaf (**A**), steam (**B**), root (**C**) in cultivars tomato treated P-stress. Columns with different capital letters compare cultivars (tolerant and sensitive) between P stress levels (control, moderate deficiency, and severe deficiency). Lowercase letters compare cultivars (tolerant and sensitive) with the same color as a function of P stress levels (control, moderate deficiency, and severe deficiency). Different letters differ by the Scott–Knott test (P < 0.05). Columns corresponding to means of six repetitions and standard deviations.

The sensitive cultivar was 8.1% more efficient in using P in the leaf (Fig. 7A), 7.9% in the stem (Fig. 7B), and 8.1% in the root (Fig. 7C) than in the control. With increasing P stress, the tolerant cultivar was higher by 8.6% in the leaf, 10% in the stem, and 8.3% in the root under moderate deficiency, while under severe deficiency it was 6% higher in the leaf, 20% in the stem, and 60% in the root.

### Physiological efficiency as a function of P and cultivar stresses

**Figure 8:**
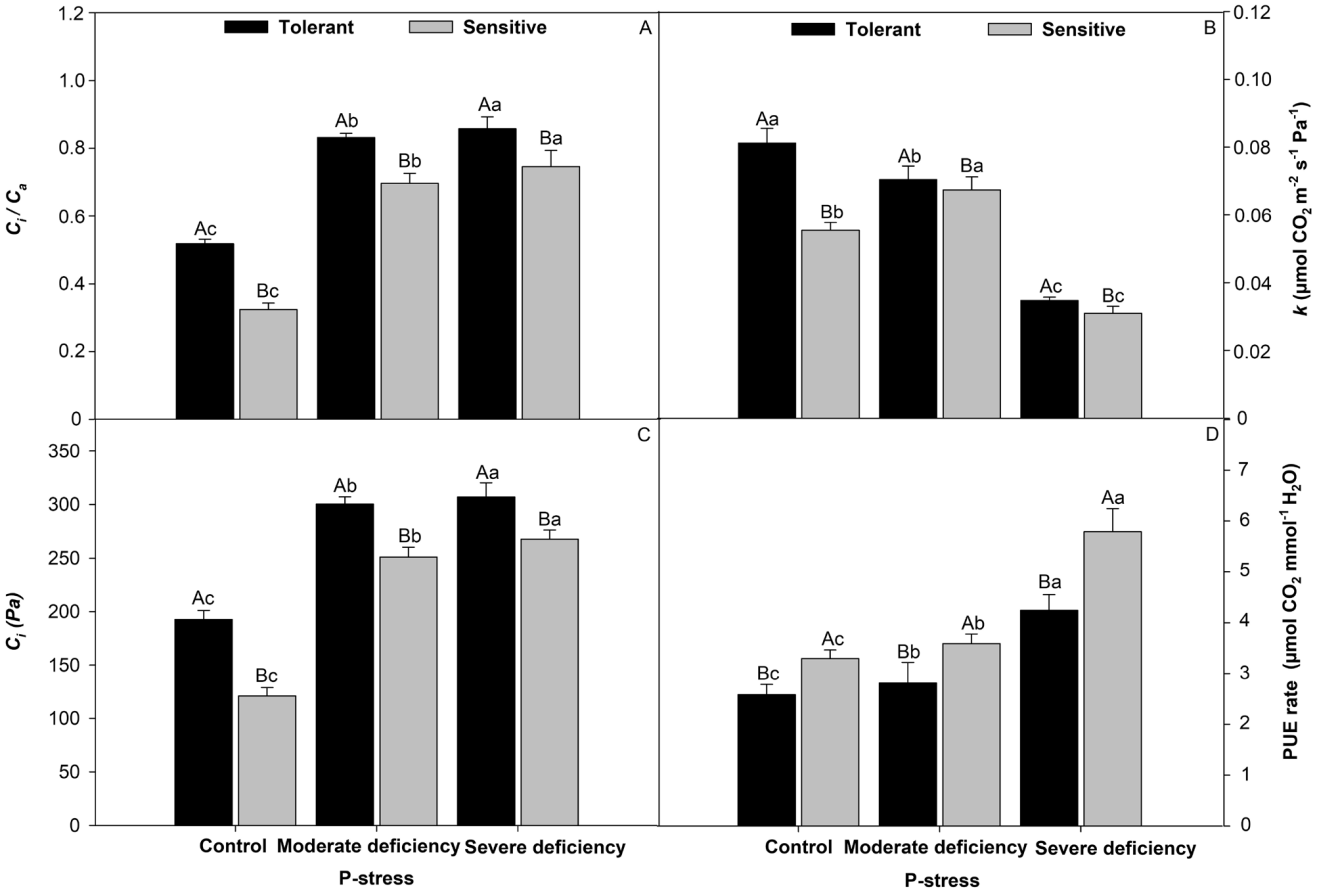
The internal carbon/atmospheric carbon ratio (Ci/Ca) refers to the relationship between the CO_2_ concentration in the leaf mesophyll and the CO_2_ concentration in the air outside the leaf, intercellular CO_2_ concentration Ci (µmol CO_2_ s^−1^ mol^−1^) (**A**), instant carboxylation efficiency (**B**), intercellular CO_2_ concentration (**C**), P-use physiological efficiency (**D**) in tomato cultivars treated P-stress. Columns with different capital letters compare cultivars (tolerant and sensitive) between P stress levels (control, moderate deficiency, and severe deficiency). Lowercase letters compare cultivars (tolerant and sensitive) with the same color as a function of P stress levels (control, moderate deficiency, and severe deficiency). Different letters differ by the Scott–Knott test (P < 0.05). Columns corresponding to means of six repetitions and standard deviations.

There was a larger ratio of *C*_*i*_/*C*_*a*_ of 62% in the control, 83% in the moderate deficiency, and 87% in the severe deficiency in the tolerant cultivar (Fig. 8A). For instant carboxylation efficiency (*K*) (Fig. 8B) of the tolerant cultivar, the values were 60% higher than those in the control, 81% higher under moderate deficiency, and 89% higher under severe deficiency. The same trend was observed in intercellular CO_2_ a larger ratio in Fig. 8C, with greater activity for the tolerant cultivar by 68% when compared to the control, 95% under moderate deficiency, and 89% under severe deficiency for different levels of P stress. Finally, for P-use physiological efficiency (Fig. 8D), the sensitive cultivar was superior in photosynthesis demand, which was 7% greater than that of the control, 7.8% under moderate deficiency, and 73% under severe deficiency, for the tolerant cultivar.

## Discussion

### Effect of P stress on dry matter production

Morphological changes include an increase in the root-shoot ratio due to limited biomass production^23^. This is the result of root growth, which is commonly maintained or increased. This improved root development is interpreted as an attempt by the plant to increase the volume of soil exploitable by the roots to better take advantage of P-enriched microdomains and to significantly increase P acquisition^24^. Phosphorus limitation plays an important role in plant growth and metabolism. Soil P generally limits plant growth owing to its poor mobility^23^. P deficiency has been reported to affect the growth, photosynthesis, and dry matter concentration of tomato plants^25^. P stress affected the dry matter mass of tomato plant leaves, stems, and roots, with greater intensity in the sensitive cultivar (Fig. 1). The effects of P deficiency on plant growth reduce photosynthesis^26^. This is because P is considered a primary nutrient for plant growth^27^ and is required to increase the production of dry matter from the roots, stems, and leaves of tomato plants^28^. Recently, there has been an rising number of published studies on the genetic, molecular, and physiological regulatory aspects of root architecture in relation to plant nutrient efficiency^29^. Root hair length is an important determinant of P acquisition, and greater root volume and root hair length have robust synergism with root hair density^30^.

### The effects of P-deficiency stress on different sizes of tomato roots

**Figure 9:**
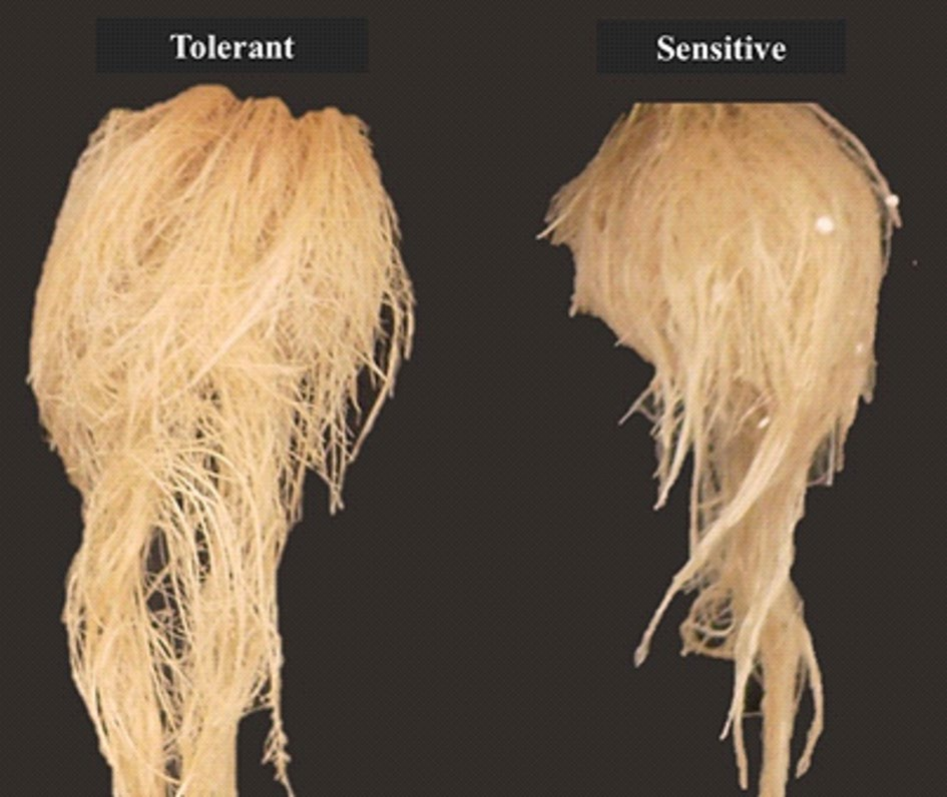
Image of the root of the cv. tolerant and sensitive in conditions of severe P-deficiency proposed by Marques et al. (2021).

The P-deficiency-tolerant tomato used in the present study showed a strong relationship between root architecture and root development. This root development is interpreted as an attempt of the plant to increase the soil explorable volume by roots for better exploitation of the P enriched micro-domains and for a significant increase of P acquisition^3^. Regardless of the P-stress levels, the tolerant cultivar had an increase in the length, surface area, and volume of the roots compared with the sensitive cultivar (Fig. 2). For all evaluated characteristics, the plasticity of roots affected by P stress is the ability of a root system to alter its typical structure in response to changing environmental conditions to acquire P at minimal metabolic costs, primarily in sensitive cultivars. As there is no universal least-cost mode of P acquisition, plasticity is important for a root system to adapt to the changing costs of adaptive strategies when external P concentrations fluctuate^31^. One of the primary modifications in plants for the acquisition of P in deficient soils is the increase in soil exploitation through greater proliferation and root growth, mainly of roots metabolically responsible for this function (roots of smaller diameter)^32^. Greater root growth of thin and very fine roots, observed in the “tolerant cultivar “Globonnie,” may be linked to this act of root exploration, which is done to obtain more phosphorus. Regarding the fineness of the root (mm), the density of the root tissue, the specific length of the root, and the diameter of the root (Fig. 3), some of the possible reasons for the tomato genotypes to have greater length, greater surface area, and a small root volume are that they favor exploration and the acquisition of water and nutrients. Changes in root morphology such as dry mass and photosynthesis can also be associated with plant growth. Thin roots play an important role in these functions^33^. Notably, under severe stress, the tolerant cultivar had much higher rates of root production than the sensitive ones, as shown in Fig. 9.

### Translocation and acquisition of P in different cultivars and P-deficiency stress levels

Phosphorus limitation plays an important role in plant growth and metabolism. Soil P generally limits plant growth owing to its poor mobility^34^. P deficiency has been reported to affect the growth, photosynthesis, and dry matter concentration of tomato plants^25^. Concerning the translocation of P to the leaves, stems, and roots (Fig. 4), the tolerant cultivar was superior in the moderate and severe treatments. Root morphology is very important in the efficient acquisition of P by plants, as the relative immobility of P makes its acquisition dependent on greater exploitation of the soil by the roots (greater length, volume, and root surface area)^35^. The root surface area is more related to the absorption of nutrients, as a larger surface area can help the plant obtain nutrients that are deficient^19^ Furthermore, an increase in the volume of roots, when the concentration of nutrients is the same on the entire root surface, can lead to a greater efficiency of nutrient absorption^36^. In an unfavorable environment, root growth (for greater exploration of the soil) is of paramount importance, without the use of costly fertilization^37^. Theoretically, a greater specific length is reflected in greater exploration efforts and better chances of acquiring water and nutrients in the soil per unit of carbon invested^35^. Therefore, the roots of tomato plants have less of the expense associated with linear construction (grams of carbon/length), as they present a greater increase in root thickness, which reflects the greater exploitation of soil with functional roots. This is because the efficiency of roots with high values of specific length can vary between species, probably because very thin roots have reduced longevity, which can prevent root function. Thus, separating the factors of root thickness (length/volume) and root tissue density (root mass/volume) can provide information regarding the relationship between root characteristics and plant development strategies^38^. After screening more than 200 tomato accessions (*Solanum lycopersicon*), at least two highly efficient introductions in the extraction of P from a nutrient solution deficient in this nutrient^39^. In one of these introductions, “tolerant cultivar Globonnie” the efficiency in the extraction of phosphorus was associated with a morphological characteristic of the roots when the access was cultivated in a nutrient solution with low P concentration. This characteristic, called “cottony root” proved to be a simple inheritance (a recessive gene, called *crt*) and is associated with a large number of roots acquired when plants grow in solutions with a low concentration (2 mg L) of P^22^. The roots were evaluated under a microscope after staining with acetic carmine. Notably, this response was not observed at higher concentrations of P (8 mg L)^22^.

### The relative leaf area and leaf area in P-stress function

Many factors influence tomato yield, of which radiation is the most important because it supplies energy for photosynthesis, which is the basic production process in plants. The leaf area index of tomatoes is influenced by stem density, number of leaves on a stem, and individual leaf size^40^. Phosphorus deficiency can negatively impact the tomato leaf area^41^ and, in cases of severe deficiency, can lead to plant death^42^. Root growth is reduced, often affecting the dry root/shoot ratio^43^, reduction in leaf production, leaf area, and light use efficiency^44^. In particular, this study found that P-deficiency reduced LAI in tomato plants by 86% (Fig. 5).

### Leaf gas exchange: photosynthesis, transpiration, and stomatal conductance

Plants have developed several mechanisms to enhance the acquisition and use of P in environments where the supply of this nutrient is limited. The efficient use of nutrients is related to the greater volume of root^22^ necessary for the acquisition of P in the soil^13^ for normal plant growth and development^19^. In our study, we observed that P-stress affected the sensitive tomato cultivar, affecting the net assimilation rate, transpiration, and stomatal conductance, normally when there is P-stress, to an immediate reduction of stomatal conductance and in parallel, a reduction of photosynthesis^45^. This effect decreases CO_2_ levels as a function of stomatal conductance, thereby reducing the internal concentration of CO_2_. The results of46 agree with our findings in this study, where P-stress affected leaf gas exchange in the P-stress-sensitive cultivar on the rates of photosynthesis, transpiration, and stomatal conductance in tomato leaves (Fig. 6). The decrease in photosynthetic activity^3^ is the result of a lack of P in soil^2^, which affects P^35^ uptake and translocation (Fig. 4) as a result of reduced root volume^22^ (Fig. 3), reduced leaf area^25^ (Fig. 5), and leaf dry mass (Fig. 1), which affects photosynthesis^26^ (Fig. 6).

### P-use efficiency of leaf, stem, and root functions under varying levels of P stress

The carbon balance depends on photosynthesis^45^ which normally decreases under P-stress conditions^19^, the instantaneous efficiency of carboxylation^50^ is closely related to the intracellular concentration of CO_2_ and the rate of assimilation of carbon dioxide. In conditions lacking P^13^, the balance between respiration and photosynthesis tends to decrease; therefore, carbon tends to decrease^26^. High values of internal CO_2_ concentration associated with an increase in stomatal conductance indicate an increase in the instantaneous efficiency of carboxylation^46^. The results of our study showed an increase in photosynthesis, transpiration, and stomatal conductance^26^ in the P stress-tolerant cultivar (Fig. 6). The reduction in photosynthetic activity resulted in decreased growth^19^ and photosynthesis^47^ in the P-stress-sensitive tomato cultivars. These results are associated with stomatal closure, which causes greater limitation of the influx of CO_2_ from the atmosphere to intercellular spaces^50^. P limitation in plants affects several physiological processes that are affected by P-stress^48^. The results of this study corroborate those found in the literature, which reported that greater root volume (Fig. 3), P translocation (Fig. 4), and leaf area (Fig. 5) can improve P^30^ absorption, favoring greater CO_2_ during carboxylation^48^.

### P-use physiological

P participates in several metallic processes, and limiting this mineral element causes changes in the photosynthetic activities of plants^48^. This element is part of the metabolism of nucleic acids, and its deficiency causes changes in plant size, leaf color tone, and root growth^11^. P deficiency induces morphological changes in the leaf architecture and plant growth^49^. Cultivated plants have high growth rates, and root morphology shows great variation in growth; when any nutrient limits, in particular, P or fixed carbon supply, the roots become drains, affecting nutrient uptake^50^. These results were observed in the P-deficient plants (Fig. 8). Roots reduce nutrient absorption during periods of nutrient scarcity^51^. Other studies have shown that deficient nutrient supply affects growth and development^52^ and that plants cannot complete their long-term cycle^53^. The physiological P-use results suggest that differences in growth and physiological characteristics of P-deficient tomato plants may have important consequences for P absorption, growth, and photosynthesis.

## Conclusion

P limitation in the tolerant cultivar promoted higher dry matter concentration (root, stem, and leaf), leaf area, root volume, nutrient translocation, rate of leaf gas exchange, and efficiency under P-deficiency stress. It was concluded from the research that the variation in the dynamics of absorption and efficiency of P use of the tolerant cultivar increased the production of roots, leaves, and leaf gas exchange under P-stress conditions.

## Methods

### Field site description

The study was carried out under greenhouse conditions in an arch-detached type structure that was 9 m wide, 25 m long, and had a 4.0 m ceiling height. A diffusing film was used for covering (140 μm in thickness), and it was characterized by its photoselectivity, antiviral properties, light-diffusing properties, anti-aesthetic properties, and resistance to ultraviolet rays^54^.

### Conducting the research

Two tomato cultivars with contrasting phosphorus absorption efficiencies were used. These were “Globonnie,” which is deficiency tolerant^19^, and “Tom-598,” which is deficiency sensitive^19^ (Fig. 9). “Globonnie” is a plant obtained from the USDA/Ames/Iowa and is freely distributed for research purposes. TOM-598 is a proprietary tomato breeding line with a standard sensitive response to P deficiency, obtained by W. R. Maluf, one of the co-authors of this paper. In the tolerant cultivar ‘Globonnie’, the efficiency of phosphorus extraction was associated with morphological characteristics associated with roots. This characteristic, called "cottony root,” was shown to be of simple inheritance (a recessive gene, called *crt*) and is associated with a large number of roots or hair roots in soils with low P concentration. Cultivar “Tom-598” is from the Santa Cruz group with a tomato background of the Santa Clara variety, characterized as sensitive to a lack of P.

The experimental protocols involving plant materials and analyses were conducted according to the institutional and international guidelines from the creators of the methods.

### Seedling production and treatments

Sowing was carried out in phenolic foam, a sterile substrate based on phenolic resin, which is free of fungi and bacteria and is typically used for rooting seedlings. The seedlings remained in the phenolic foam for 30 d and then transplanted into 10 L pots. Within these structures, the vessels were placed on benches for 15 d after transplanting. The tolerant and sensitive cultivars were grown in three nutritive solutions, with the only variation being the P concentration. The pH was standardized to 5.5 ± 0.5, using NaOH 0.1 M L^−1^ solution, the electrical conductivity was maintained at 2.5 dS m^−1^, and the nutrient solution was constantly oxygenated^55^. The final evaluation was performed 150 days after transplantation.

### Experimental design

The experimental design consisted of a factorial scheme composed of two tomato cultivars: tolerant and sensitive to P deficiency, and three levels of P (control, moderate deficiency, and severe deficiency). The P concentration was characterized as control (60 mg L^−1^), moderate deficiency (30 mg L^−1^), and severe deficiency (15 mg L^−1^ of P_2_O_5_), as proposed^55^. Each plot consisted of one plant per pot with six replicates per plot.

### Preparation of root, stem, and leaf samples

For the analysis of the root system, whole plants were collected. Thereafter, the root, stem, and leaves were separated. The samples were placed in a tray containing distilled water for 30 min. After the roots were washed with distilled water and placed in hermetically sealed plastic pots, 70% ethanol solution, stored in a refrigerator at 2 ± 2 °C, was added to the pots.

### Root, stem, and leaf dry matter

Whole plants were collected for root system analysis. The roots, stems, and leaves were then separated. The samples were then placed in a tray containing distilled water for 30 min. After the roots were washed with distilled water and placed in hermetically sealed plastic pots, 70% ethanol solution stored in a refrigerator at 2 ± 2 °C was added to the pots.

### Analysis of root system morphology and dry mass attributes

The WinRHIZO Pro 2007 system (Regent Instruments, Sainte-Foy, QC, Canada) was used to analyze root system morphology. This was coupled with a professional Epson scanner (Expression 10,000 XL; Epson America, Inc., USA) equipped with an additional light unit (TPU). A definition of 400 (dpi) was used for root morphology measurements^36^. The roots were placed in an acrylic tube (20 cm wide × 30 cm long) containing water. The use of this accessory made it possible to obtain images in three dimensions and prevent root overlap. Six samples were analyzed for each tolerant and sensitive cultivar. The following characteristics were determined: root volume (mm^3^), root surface area (mm^2^), root length (mm), root fineness (mm mm^−3^), root tissue density (g mm^−3^), specific root length (mm g^−1^), average root diameter (mm) of tomato plants. Other attributes involving dry mass morphological data were specific length (the relationship between length and the average diameter of the root) and root fineness (the relationship between length and volume of the root).

### Leaf area of tomato cultivars

The leaf area of the tomato plants was quantified using an LI-COR 3000 leaf area meter. The length was measured from the tip of the blade to the point of intersection of the blade and petiole, and the width was measured from end to end, between the widest lobes of the blade. The relative leaf area (RLA) was calculated by dividing the area of each leaf by the average leaf area of the plant, as follows: relative leaf area (RLA), leaf area (LA) of a given leaf (cm^2^), and average leaf area (LAM) of the plant (cm^2^ leaf^−1^), given by the sum of the LA of all leaves, divided by the number of leaves of each plant.

### Leaf gas exchange and physiological relations in tomato plants

Leaf gas exchange, net assimilation (*A*), transpiration (*E*), stomatal conductance (*gs*), intercellular CO_2_ concentration, P-use physiological efficiency, and instant carboxylation efficiency were evaluated using IRGA model LI-6400XT (Li-Cor, Lincoln, Nebraska, USA). Six plants were randomly obtained from each plot, and the sample unit was represented by the sixth leaf count from the top, which was completely expanded and mature. Because it is a species with compound leaves, the first three leaflets of each leaf were used in the measurements, totaling six measurements. Saturation irradiance was fixed at 850 μmol m^−2^ s^−1^, which is the value defined by a light curve that induced the maximum rate of photosynthesis. Tomato is a C3 plant, whose cyclic mechanism of enzymatic reactions converts CO_2_ into carbohydrates through the reductive photosynthetic cycle (C3), generating 3-phosphoglycerate. For this reason, the temperature of the IRGA camera was controlled at 28 °C because the maximum photosynthesis rate is achieved at a relatively low radiation intensity, causing no destruction or damage to the photosynthetic apparatus. Measurements were performed on a sample of leaves with an area of 6 cm^2^. *P*_*n*_–C_a_ and *P*_*n*_–*C*_*i*_ curves were obtained under saturation (850 μmol m^−2^ s^−1^) using a gas extender (ADC, Hoddesdon, UK) coupled to an IRGA camera (LI-6400XT). It was used to adjust the *P*_*n*_–*C*_*a*_ and *P*_*n*_–*C*_*i*_ curves by exchanging the component with the CO_2_ concentration. The potential photosynthetic capacity, *P*_*nmax*_ (μmol m^−2^ s^−1^), was obtained using a modified version of Equation (I). Equation (II) was used to obtain the apparent efficiency of carboxylation using the *P*_*n*_–*C*_*i*_ curve, as recommended^56^.

### Nutritional determination in tissues

Samples of the root, stem, and leaf were analyzed for mineral composition at day 105 after planting. The analyses were conducted in the laboratory of mineral nutrition of plants in the Department of Soil Science, Federal University of Lavras, UFLA, Brazil, and the methodologies used are described^57^.

### P stress index

To determine the P-use efficiency (PUE), we used the equation PUE = (P-acquisition efficiency × P-utilization efficiency), where the P-acquisition efficiency (PAE) level of P is expressed in mg kg and the P-utilization efficiency is expressed in mg kg. To determine the P-acquisition efficiency, we used the equation PAE = (P-total dry matter/P-amount available in solution), where P-total is the amount of P expressed in dry matter and P is the P available in a solution of P. To determine the P-utilization efficiency (PUtE), we used the equation PUtE = (total dry matter/P-total dry matter) recommended^58^.

### P concentration and translocation

The P concentration in leaves was expressed as percentages [(mass of nutrient/dry mass of organ) × 100] and in terms of mg kg. To determine the total concentration of P in each vegetative organ, we used the equation NC = (NL/100) × DM, where NC is the concentration of P, NL is the level of P expressed as a percentage, and DM is the leaf dry mass. The results are expressed in grams (g). To determine nutrient translocation (transport from root to shoot), we used the equation NT = (NC/TNP) × 100, where NT represents nutrient translocation, NC is the nutrient mass, and TNP is the total nutrient concentration of the plant. The results were expressed as percentages, as previously described^59^.

### P-physiological efficiency

The efficiency of the absorption of P (PUE), defined as the product of the efficiency of acquisition and internal use of P, was estimated according to the dry mass of the leaflets, stems, and roots produced per kg of phosphorus^58^.

### Statistical analysis

The data were subjected to analysis of variance, and when significant differences occurred, the Scott-Knott test at a 5% probability of error was applied. Standard errors were calculated for all the media. All statistical analyses were performed using the SAS software version 9.3^60^.
